# Lizard Body Temperature Acquisition and Lizard Recognition Using Artificial Intelligence

**DOI:** 10.3390/s24134135

**Published:** 2024-06-26

**Authors:** Ana L. Afonso, Gil Lopes, A. Fernando Ribeiro

**Affiliations:** 1 Mechanical Engineering Department, University of Minho, 4800-058 Guimarães, Portugal; ana.leonor.afonso@gmail.com; 2 LIACC, University of Maia, 4475-690 Maia, Portugal; alopes@umaia.pt; 3 Centro ALGORITMI, University of Minho, 4800-058 Guimarães, Portugal

**Keywords:** artificial intelligence, body temperature acquisition, computer vision, lizards, object detection, YOLO

## Abstract

The acquisition of the body temperature of animals kept in captivity in biology laboratories is crucial for several studies in the field of animal biology. Traditionally, the acquisition process was carried out manually, which does not guarantee much accuracy or consistency in the acquired data and was painful for the animal. The process was then switched to a semi-manual process using a thermal camera, but it still involved manually clicking on each part of the animal’s body every 20 s of the video to obtain temperature values, making it a time-consuming, non-automatic, and difficult process. This project aims to automate this acquisition process through the automatic recognition of parts of a lizard’s body, reading the temperature in these parts based on a video taken with two cameras simultaneously: an RGB camera and a thermal camera. The first camera detects the location of the lizard’s various body parts using artificial intelligence techniques, and the second camera allows reading of the respective temperature of each part. Due to the lack of lizard datasets, either in the biology laboratory or online, a dataset had to be created from scratch, containing the identification of the lizard and six of its body parts. YOLOv5 was used to detect the lizard and its body parts in RGB images, achieving a precision of 90.00% and a recall of 98.80%. After initial calibration, the RGB and thermal camera images are properly localised, making it possible to know the lizard’s position, even when the lizard is at the same temperature as its surrounding environment, through a coordinate conversion from the RGB image to the thermal image. The thermal image has a colour temperature scale with the respective maximum and minimum temperature values, which is used to read each pixel of the thermal image, thus allowing the correct temperature to be read in each part of the lizard.

## 1. Introduction

Lizards are ectothermic animals, which means that they do not produce enough metabolic heat to maintain their body temperature, having to resort to the use of external heat sources. In biology laboratories, measuring body temperature in lizards can provide relevant information to biologists. Traditionally, the body temperature of lizards is measured using a contact thermometer. This method is extremely invasive and painful for the animal. Also, it is impossible to obtain the temperature from different lizard body parts.

Thus, a new method emerged that consisted of filming the lizard kept in captivity with a Forward-Looking Infrared Camera (FLIR), also known as a thermal camera. Later, using specialised software for this type of camera (in this case, FLIR Tools), temperatures of the body parts of the animal under study were obtained by manually clicking on each of the body parts in the video and recording their value. This process is carried out every 20 s of the video. The entire process does not occur in real time. There is also a possible loss of information regarding changes in the lizard’s body temperature between each measurement process.

This new method proved to be advantageous for the animal, as it is not an invasive method. However, the entire procedure of obtaining temperature values in different parts of the body follows a time-consuming, difficult, monotonous, and not very rigorous method (for example, due to potential inaccuracies when manually clicking on parts of the lizard’s body). Therefore, it is desirable to overcome the adversities presented by this new measurement method. To this end, a different approach is proposed. By using artificial intelligence and a combination of an RGB camera and a thermal camera, it is possible to detect the lizard and its body parts automatically and obtain the respective desired body temperature values quickly and coherently. This system can be applied to images of previously recorded videos. In both cases, the final values are automatically saved into a text file. In addition, a greater flow of data allows more detailed maintenance of the lizards, and the time that was spent by biologists in manually obtaining measurements can be used for other purposes.

The method presented in this paper provides biologists with a faster and non-intrusive way to measure the temperatures of lizards placed in a box in a controlled laboratory setting. In these controlled environments, different temperatures can be applied to various sections of a box, allowing researchers to monitor the temperature preferences of lizards as they choose where to move to get warmer. This capability is crucial for studying the behavioural responses of lizards to temperature changes, enabling detailed observations of their thermoregulation strategies.

The significance of this research lies in its contribution to more efficient and humane methods of monitoring lizard body temperatures, which are essential for understanding their behaviour and physiological needs. By automating the temperature acquisition process, our method reduces the stress and potential harm to the animals, providing a more ethical approach to studying their behaviour. Additionally, the insights gained from such studies can inform broader ecological research and conservation efforts, particularly in understanding how lizards might adapt to changing environmental conditions.

### 1.1. Artificial Intelligence

Artificial intelligence (AI) speeds up human tasks with a guaranteed level of precision and accuracy. With the emergence of new algorithms, the progress in computing power and storage, and the accessibility to a vast quantity of data, AI suffered notable breakthroughs and is already being applied to numerous fields, such as the field of biology.

Researchers are regularly confronted with complex and time-consuming problems. Thus, AI emerges to offer solutions to these problems and promote innovation in laboratories. Biological research and artificial intelligence are becoming increasingly related. Developing tools for the analysis and interpretation of vast amounts of data is one of the most significant uses of artificial intelligence in biology. AI is already present in a variety of biology research works, such as:

- Protein 3D structure prediction: AI helps predict the three-dimensional structure of proteins and subsequently understand their function, enabling the development of new specialised drugs [1].
- Drug development: AI helps speed up drug development [2].
- Conservation and wildlife tracking and monitoring: AI helps protect wildlife and natural resources and helps automate wildlife tracking and monitoring [3].

### 1.2. Machine Learning and Deep Learning

Machine learning (ML) is a subset of AI that aims to give a computer the ability to learn from experience, using data instead of being explicitly programmed. An ML model is the output generated after training the ML algorithm with data [4]. Supervised learning (SL) is one of the main ML approaches, where a set of labelled training data, sample data (input), and associated target responses (output) are provided to the algorithm for it to learn a function that maps an input to an output, and a predictive model is created [5]. This model is then used to make predictions on never-seen samples.

The SL algorithm needs to have the capability of generalising from training data to unseen samples. The model testing should not be carried out on the training data because it gives the false impression of success; instead, it should be carried out on new examples.

Overfitting and underfitting are two common problems in ML. Overfitting occurs when the model can predict correctly all the labels of the training data but does not generalise well to unseen data; in this case, the model has a low bias and a high variance (high complexity model) [6]. On the other hand, underfitting occurs when the model cannot generalise well to unseen data and makes mistakes trying to predict the labels of the training data; in this case, the model has a high bias and a low variance (low complexity model) [6]. Overfitting and underfitting can occur due to several reasons, such as an inadequate size and quality of the training dataset.

Bias represents how closely the average prediction is to the true value, and variance quantifies how much, on average, predictions vary for different sets of training data [7]. To obtain the ideal model, it is essential to find the optimal balance between bias and variance.

Deep learning (DL) is a subset of ML based on neural networks. Neural networks are inspired by the structure of the human brain and the way it works and consist of three types of layers: the input layer, the hidden layer, and the output layer. An Artificial Neural Network (ANN) is a type of neural network with one or two hidden layers. A Convolutional Neural Network (CNN) is a type of ANN.

CNNs were first introduced with the design of LeNet-5 by Yann LeCun et al. [8] The introduction of Graphics Processing Units (GPUs), faster Central Processing Units (CPUs), and the increasing amount of training data available have driven the development of new architectures, such as AlexNet [9], ZFNet [10], GoogLeNet [11], ResNet [12], VGGNet [13], and EfficientNet [14], as well as several object detectors based on CNNs, including the R-CNN (Region-Based Convolutional Neural Networks) family of two-stage detection networks and the one-stage detection networks SSD (Single Shot MultiBox Detector) [15], RetinaNet [16], EfficientDet [17], and the YOLO (You Only Look Once) family.

Hao et al. [18] proposed a lightweight detection algorithm based on the one-stage detection network SSD for sheep facial identification, achieving a mAP of 83.47% and a detection speed of 68.53 frames per second. Jia et al. [19] developed a marine organism object detection model also based on a one-stage detection network, the improved EfficientDet, obtaining a mAP of 91.67% and a processing speed of 37.5 frames per second.

Roy et al. [20] presented a comparative study between the one-stage detection networks RetinaNet, SSD, YOLOv3, and YOLOv4 and the two-stage detection networks Mask R-CNN and Faster R-CNN for wildlife detection. The findings indicated that YOLO variants outperformed the other networks, with the one-stage detection network YOLOv4 achieving the best performance (mAP of 91.29%). Hu et al. [21] conducted a study utilising Detectron2, RetinaNet, YOLOv4, and YOLOv5 models to determine the count of cattle in satellite images, with YOLOv5 achieving the best results, producing an average precision of 91.60% and a recall of 91.20%. Both studies by Roy et al. [20] and Hu et al. [21] demonstrate the effectiveness of the YOLO family in animal detection.

Jubayer et al. [22] found that the overall performance of YOLOv5 in detecting mould on food surfaces was superior to that of YOLOv4 and YOLOv3, achieving an average precision of 99.6%. Long et al. [23] developed a system for fish detection, where YOLOv5 also obtained the highest mAP value of 95.95%, superior to YOLOv3 and YOLOv4. Ahmad et al. [24] conducted a study comparing the performance of YOLO-Lite, YOLOv3, YOLOR, and YOLOv5 in identifying insect pests, with YOLOv5 emerging once more as the most successful, achieving an average precision of 98.3%.

### 1.3. Current Research Status

The automated detection of animals and the extraction of body temperature values play critical roles in various domains within animal studies.

Advances in deep learning have stimulated the growth of studies focused on the automatic detection of animals for various purposes, such as forest wildlife monitoring and conservation [25], agriculture and farming [26,27], and species identification and classification [28,29]. While most studies on automatic animal detection predominantly focus on mammals and birds, studies addressing reptiles, particularly lizards, are relatively scarce. Aota et al. [30] addressed this gap by developing a deep neural network-based system for detecting the invasive lizard species *Anolis carolinensis* in drone images. This study aims to contribute to an effective and efficient approach to conserving ecosystems, as this invasive species threatens the native insect population of the Ogasawara Islands in Japan.

The body temperature of an animal is a crucial indicator of its health and well-being. However, traditional methods for obtaining these values are challenging. Consequently, there has been a notable increase in studies dedicated to developing automated methods for temperature extraction in animals. A substantial portion of these studies focuses on obtaining temperature data to monitor and assess the health status of pigs and cows [31,32]. Conversely, there is a notable scarcity of studies concerning the automated extraction of body temperature in lizards.

This paper addresses this research gap by developing a system capable of automatically detecting lizards and their body parts using YOLOv5s, followed by the automatic and contactless extraction of temperature values from the detected parts. This system allows biologists to easily obtain valuable data on the body temperature of lizards to use in their research without causing pain or stress to the animal. Karameta et al. [33] obtained the body temperature of insular agamid lizards by inserting a type K thermocouple directly into the animal’s cloaca to study how seasonality impacts the thermal biology of an island population of lizards, providing insights into their survival strategies and potential adaptations to future environmental changes. The use of a non-invasive (contactless) and automatic system to extract these temperature values, such as the one developed in this paper, would have been a huge advantage in this study.

Furthermore, the system developed in this article offers the potential to be adapted and adjusted to extract the body temperature of various species of lizards and other reptiles.

## 2. Methodologies

This work presents a system capable of detecting the entire lizard and six pre-defined parts of its body (snout, head, back, left leg, left palm, and tail) in an image or a video and then displaying and recording the temperature values in these regions. It consists of two main parts: the development of a model for detecting the lizard and its body parts and the acquisition of the temperature values of the detected parts. All algorithms were developed in the Python language and supported with the OpenCV library.

### 2.1. Detection of Lizard Body Parts

Detection of lizard body parts was developed using the YOLOv5 ML algorithm.

#### 2.1.1. YOLOv5

Object detection is a task focused on localising and classifying objects present in images or videos.

YOLO (You Only Look Once) is a state-of-the-art, real-time object detection algorithm. The fifth version of YOLO (YOLOv5) was proposed in 2020 by the company Ultralytics and is the version selected to use in this project, taking into account the YOLOv5 detection accuracy and detection speed. It is important to note that at the time of the practical development of this paper, YOLOv5 was the current version in use; therefore, later versions were not considered.

**Figure 1:**
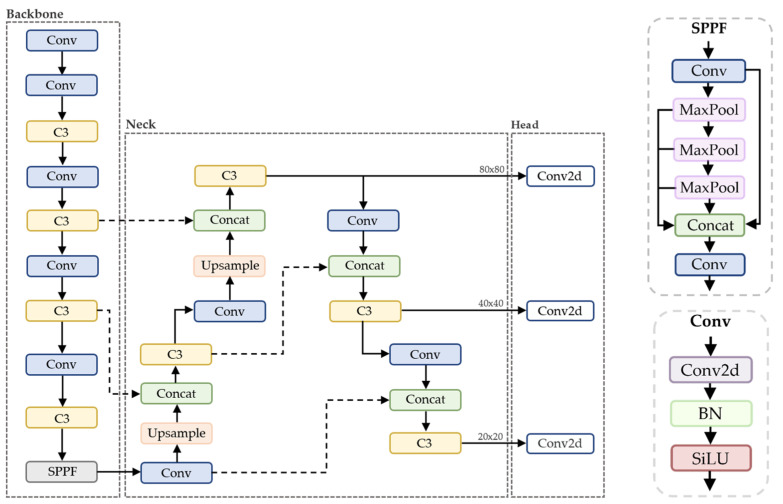
Architecture diagram of YOLOv5s.

The YOLOv5 architecture is composed of three parts: CSP-Darknet53 as the backbone, Spatial Pyramid Pooling Fusion (SPPF) and CSP-PAN (Path Aggregation Network) structures in the neck [34], and the same head as YOLOv3. CSP-Darknet53 is formed by applying a Cross Stage Partial Network (CSPNet) to Darknet-53. The amount of computation may be significantly decreased with CSPNet, and both the inference speed and accuracy can be improved [35]. In the neck, the SPPF is a faster variation of a Spatial Pyramid Pooling (SPP) block. Figure 1 shows the architecture diagram of YOLOv5s.

Contrary to previous versions, YOLOv5 uses the PyTorch framework instead of the Darknet framework [36]. To reduce overfitting and improve the model’s ability to generalise, YOLOv5 uses some data augmentation techniques, such as mosaic augmentation.

YOLOv5 is divided into five different model sizes: YOLOv5n (nano), YOLOv5s (small), YOLOv5m (medium), YOLOv5l (large), and YOLOv5x (extra-large). Larger models contain more parameters, need more memory to train, require larger and well-labelled datasets, and take longer to execute but will generally produce better results. On the other hand, smaller models are faster but may abdicate some accuracy.

To evaluate the performance of a certain object detection model, some metrics are used, such as intersection over union (IoU), confusion matrix, precision (P), recall (R), F1 score, average precision (AP), and mean average precision (mAP).

The intersection over union metric estimates how well a predicted bounding box matches the ground truth bounding box and is given by a ratio between the intersection area (area where the boxes overlap) and the union area (total area of both boxes) of the predicted bounding box with the ground truth bounding box.

A confusion matrix is a table in which the values predicted by the classifier are compared with the ground truth labels. This table is composed of four types of predictions: false positive (FP), false negative (FN), true positive (TP), and true negative (TN).

Precision counts the percentage of predicted positives that are actually positive and is calculated using Equation (1). Recall measures the percentage of positives correctly detected and is calculated using Equation (2). The F1 score combines precision and recall and ranges between 0 and 1. The F1 score is obtained using Equation (3).

(1) $$Precision=\frac{Correct\;Predictions}{Total\;Predictions}=\frac{TP}{TP+FP}$$

(2) $$Recall=\frac{Correct\;Predictions}{Total\;Ground\;Truth}=\frac{TP}{TP+FN}$$

(3) $$F1score=2\times \frac{Precision\times Recall}{Precision+Recall}$$

The area under the PR curve (AUC) gives the average precision (AP) and is calculated using Equation (4). The mean average precision (mAP) is obtained by taking the mean of the average precision obtained in every class, as shown in Equation (5).

(4) $$AP=\int_{r=0}^{1}p\left(r\right)dr$$

(5) $$mAP=\frac{1}{N}\sum_{i=1}^{N}{AP}_{i}$$

#### 2.1.2. Selection of YOLOv5 Model Size

Initially, to choose the ideal YOLOv5 model size for the required application (detection of specific body parts of a lizard), training and inference were carried out for each one of the YOLOv5 model sizes under the same conditions.

An RGB dataset containing 10288 images was initially created from scratch to be later used in training. For training, 100 epochs and a batch size of 16 were used.

Table 1 and Table 2 show the values obtained for precision, recall, mAP, training duration, number of parameters, GFLOPs (Giga Floating-point Operations Per Second), and inference time (time each model took to analyse a new image and make a prediction) using YOLOv5n, YOLOv5s, YOLOv5m, YOLOv5l, and YOLOv5x.

To select the most suitable model for the application under analysis, the best balance between speed and accuracy was sought. Although YOLOv5n was the fastest and lightest model, its results were the lowest and, therefore, the model was disregarded (Table 1). The heaviest models, YOLOv5l and YOLOv5x, obtained the best results for the evaluation metrics; however, they took a long time to complete the training (more than 10 h) and presented a higher inference time, which is a major obstacle due to time limitations. Therefore, these models were also disregarded (Table 2). Finally, both YOLOv5s and YOLOv5m models obtained good results for the evaluation metrics. Since the difference between the values of the metrics obtained for each of these models was not very significant, the YOLOv5s model was chosen as it is lighter, leading to faster training and shorter inference time.

#### 2.1.3. RGB Image Dataset

An RGB image dataset was created from scratch based on custom data. All filming took place in a controlled environment at CIBIO (Centre in Biodiversity and Genetic Resources), University of Porto, Portugal. 

**Figure 2:**
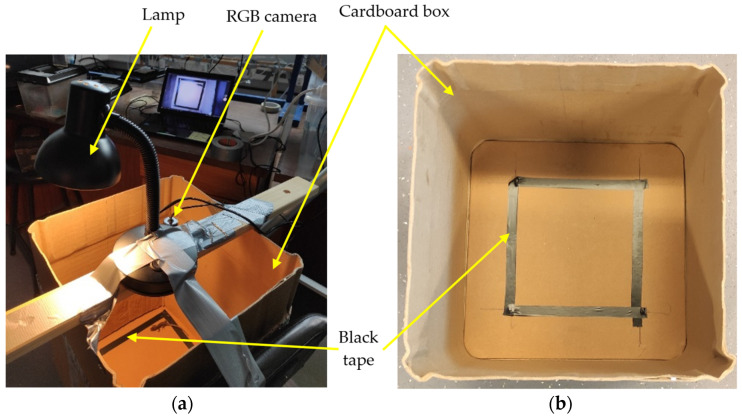
Scenario: (**a**) setup and (**b**) cardboard box used.

Firstly, a scenario was built consisting of a cardboard box, a lamp, a camera, and some black tape (Figure 2).

The lizard was placed inside the cardboard box, and the camera filmed its behaviour for about 10 min. In total, about 10 videos were collected using animals with different body sizes, colours, and patterns. All RGB images that compose the dataset were obtained from those videos, making a dataset of 4306 RGB images.

**Figure 3:**
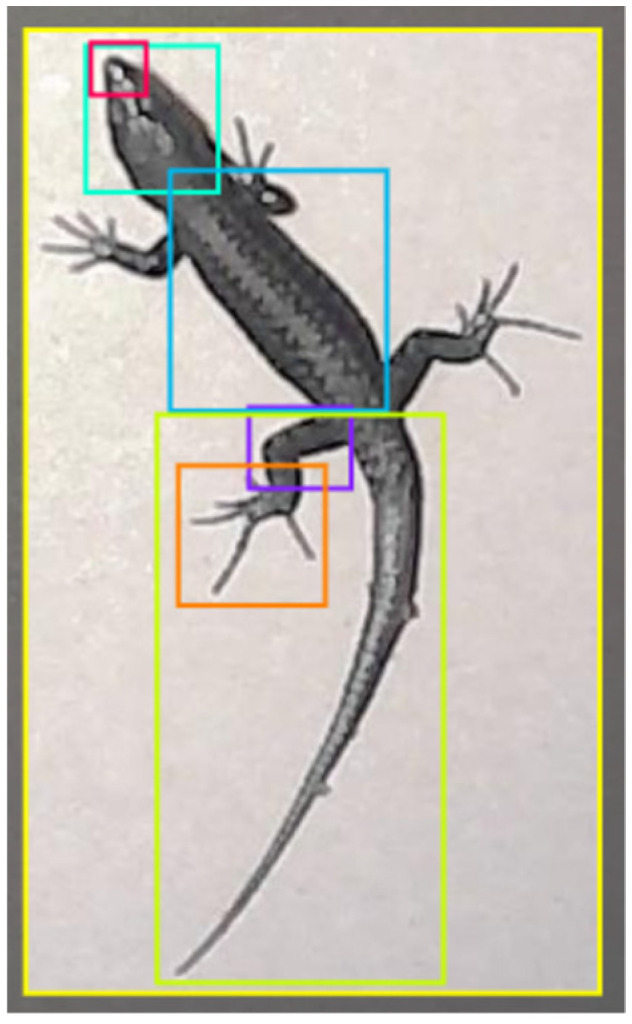
Example of a labelled dataset image in Roboflow.

The image labelling was carried out using Roboflow. For each image, bounding boxes were drawn around each part of the lizard’s body to be identified and labelled with the respective class. In total, seven classes were identified: “Lizard” (yellow bounding box in Figure 3), “Snout” (red bounding box in Figure 3), “Head” (cyan bounding box in Figure 3), “Dorsum” (blue bounding box in Figure 3), “Tail” (green bounding box in Figure 3), “Leg_L” (purple bounding box on the left hind leg in Figure 3), and “Palm_L” (orange bounding box on the left hind palm in Figure 3).

In Roboflow, inside the dataset, the images were split into three sets:

- “Training set”: is used to train the model.
- “Validation set”: is used during training to compute the validation mAP after each epoch. It is also used to evaluate the performance of the trained model.
- “Test set”: is used to analyse the final performance of the model.

The “training set” contained 3014 RGB images (70%), the “validation set” contained 861 RGB images (20%), and the “test set” contained 431 RGB images (10%).

All images were resized to 640 × 640 as it is YOLOv5’s default size, and some augmentation techniques were applied to the “training set” images to create new examples to use in the training of the model. The techniques used in the training images were modifications in saturation (between −10% and +10%), brightness (between −10% and +10%), exposure (between −10% and +10%), blur (up to 1 pixel), and noise (up to 1% of pixels). After augmentation, the dataset went from 4306 RGB images to 10,334 RGB images.

#### 2.1.4. Training and Inference

All training was carried out on Google Collaboratory, which runs in the cloud, and the NVIDIA Tesla T4 GPU (16 GB of memory) was used. Firstly, training was performed for the number of epochs and batch sizes represented in Table 3 to find the model with the best training results.

Secondly, inference was run on some images, and two thresholds were defined:

- Confidence threshold: Defines the minimum score the model considers the prediction to be correct; otherwise, it completely discards the prediction. This threshold was set to 0.50, meaning all predicted bounding boxes with a confidence score below 50% were discarded. This value was chosen based on a careful analysis of the results obtained using different threshold values.
- IoU threshold: Defines the minimum overlap between the predicted bounding box and the ground truth bounding box for the prediction to be considered correct. This threshold was set to 0.50 after a careful analysis of the results obtained using different threshold values.

The training and inference results are shown in Section 3.1.

### 2.2. Temperature Acquisition

After detecting the lizard’s position, its temperature acquisition was then possible to be acquired as described next.

#### 2.2.1. Thermal and RGB Image Acquisition

**Figure 4:**
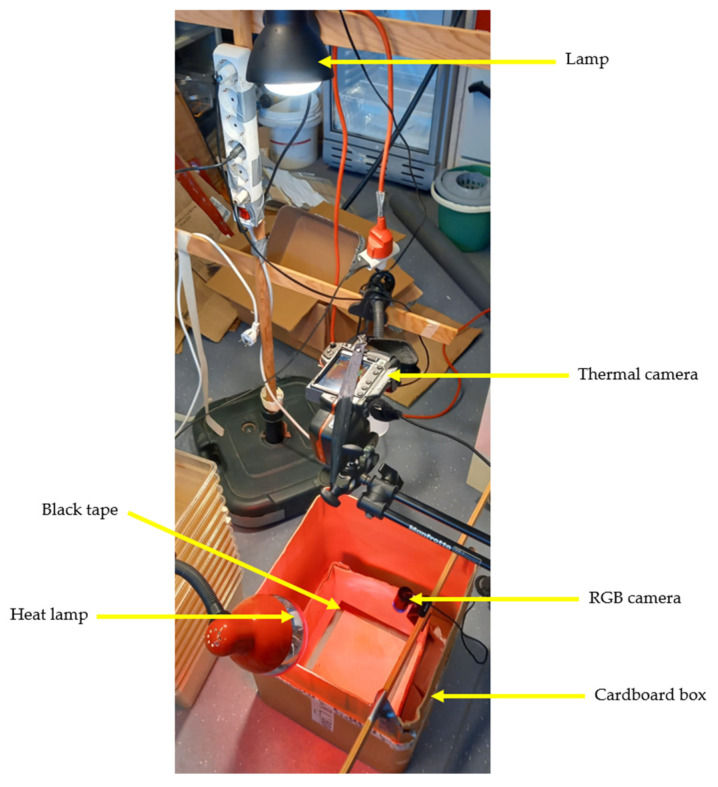
The scenario used to obtain thermal images and their associated RGB images.

To obtain the thermal images used in this work, the FLIR T335 thermal camera was added to a scenario similar to the one described in Section 2.1.3. As shown in Figure 4, the thermal camera was positioned above the RGB camera with a certain horizontal offset to try to match the point of view of both cameras as much as possible.

**Figure 5:**
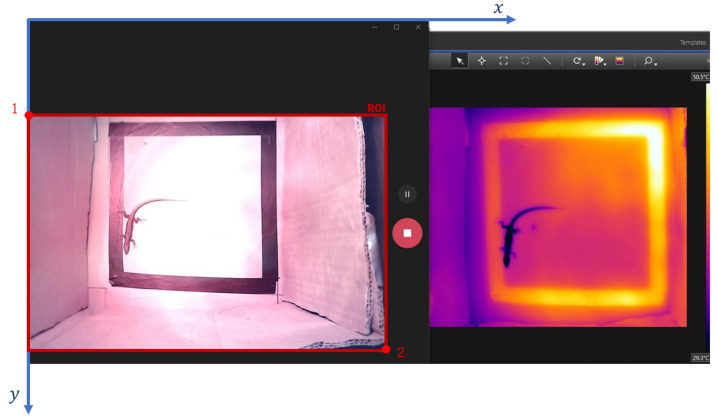
RGB camera output (**left side**) and thermal camera output (**right side**).

The lizard was placed inside the cardboard box, and both cameras simultaneously filmed the animal’s behaviour for a few minutes. The RGB camera and the thermal camera were placed side-by-side, as presented in Figure 5, and the videos were saved in the same way (screen recording). Some videos were recorded with the heat lamp on and others with the heat lamp off to observe more significant changes between videos in the animal’s colour in the thermal images (change in the animal’s body temperature). Using the RGB camera helps to determine the position of the lizard in the thermal camera, especially if the lizard is at the same temperature as the background.

#### 2.2.2. YOLOv5s Model Application

To detect the lizard and its six body parts, the model analysed in Section 3.1 was used. Since it was desirable to apply detection only to the RGB image, a region of interest (ROI) involving only the RGB image was created. The region of interest was defined using Equation (6).

ROI = image [y: y + height, x: x + width](6)

where, from Figure 5’s coordinate axes:

- “image” represents the input image, with the RGB and thermal images side-by-side;
- “x” is represented by the x-coordinate of point 1 in Figure 5;
- “y” is represented by the y-coordinate of point 1 in Figure 5;
- “y + height” is represented by the y-coordinate of point 2 in Figure 5;
- “x + width” is represented by the x-coordinate of point 2 in Figure 5.

**Figure 6:**
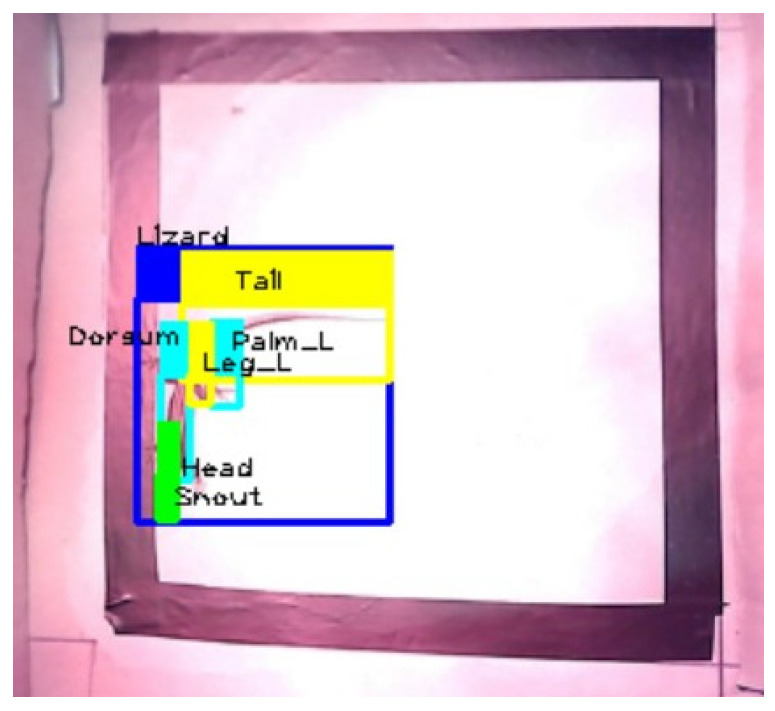
Detection of the lizard and its six body parts on the ROI (left).

Figure 6 shows the detection of the lizard and its six body parts in the defined region of interest.

The detections were only made for the RGB image and not for the thermal image, as the model was trained only with RGB images and not with thermal images. Using the model to detect the lizard and its body parts in thermal images would generate erroneous detections. Following this, the process is described with two examples: the whole lizard and its tail.

#### 2.2.3. Bounding Box: Identified Class and Background

**Figure 7:**
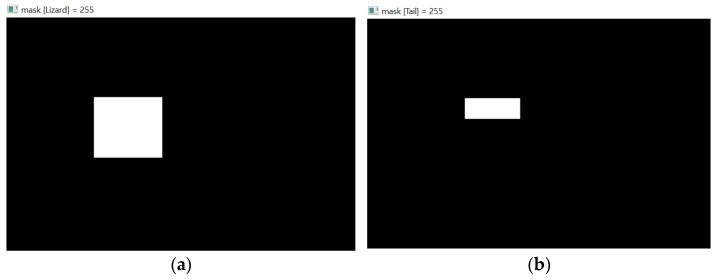
All pixels within the (**a**) “Lizard” and (**b**) “Tail” bounding boxes are white.

**Figure 8:**
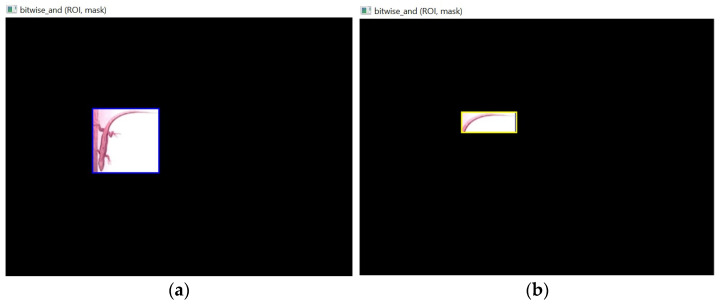
Isolation of the ROI defined by the (**a**) “Lizard” and (**b**) “Tail” bounding boxes.

**Figure 9:**
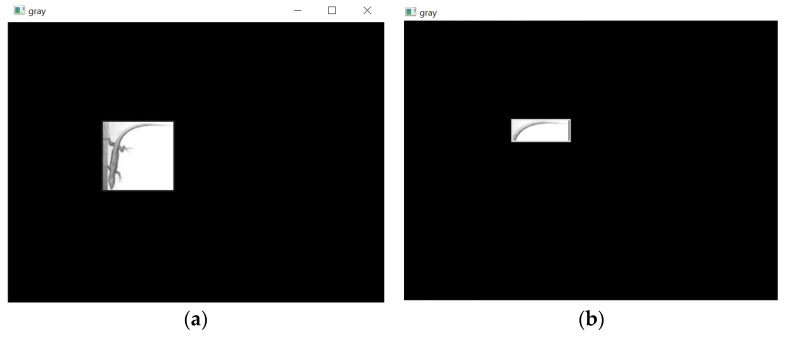
The (**a**) “Lizard” and (**b**) “Tail” bounding boxes are in grayscale.

**Figure 10:**
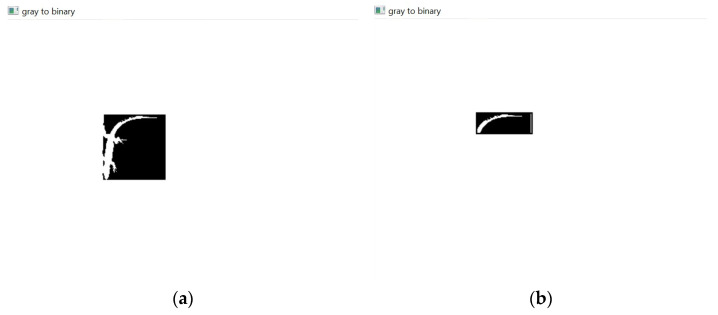
The (**a**) “Lizard” and (**b**) “Tail” bounding boxes are in black and white (binary).

After the detection process, the bounding boxes generally involve the detected class and part of the background. To make the distinction between the background and the identified class clear, the following method was used, involving five sequential steps:

1. Creation of a black binary mask with the same dimensions as ROI.
2. In the black binary mask created in Step 1, all pixels within the region of each bounding box are set to white, as shown in the examples in Figure 7.

3. Application of a bitwise AND operation between the ROI and the binary mask from Step 2. This retains only the pixels that both have non-zero values (Figure 8), which are the pixels that fall within the bounding box.

4. Conversion to grayscale, as shown in Figure 9.

5. Conversion from grayscale to binary using an inverse-binary threshold (Figure 10). This is user threshold-dependent since the threshold value must be chosen by the user.

As demonstrated in Figure 10, inside each bounding box, the background pixels turned black, and the pixels of the class to be identified turned white. This allowed us to not only highlight the most important part within each bounding box (identified class) but also make it possible to distinguish between the lizard (white pixels) and the background (black pixels).

**Figure 11:**
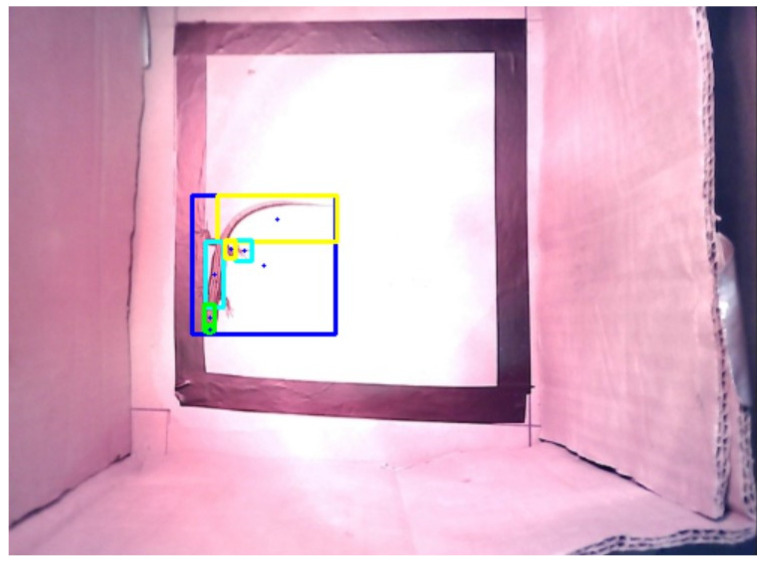
Bounding boxes and their respective central pixels are represented by a blue circle (in the ROI).

A single pixel was selected to represent each bounding box based on what was discussed and decided by the biologists. The main requirement was that in each bounding box, the pixel had to belong to the detected class, not the background. For this purpose, the pixel in the centre of each bounding box was initially considered (Figure 11).

However, as can be seen in Figure 11, not all pixels in the centre of the bounding boxes belong to the detected class, as some belong to the background. Undesirably, the pixel in the centre of the “Lizard” and “Tail” bounding boxes belonged to the background and not to the respective class.

To solve this problem, after using the method explained at the beginning of this section, a condition was created in which it was determined whether the central pixel in each bounding box was white (if center_pixel == 255) or not (else:). If it is determined that the pixel is white, that pixel would represent the bounding box; otherwise, it would search for the nearest white pixel to the central pixel (determined initially), and that would be the new pixel that should represent the bounding box. To find the coordinates of the nearest white pixel, a function called “nearest_white_pixel” was defined.

**Figure 12:**
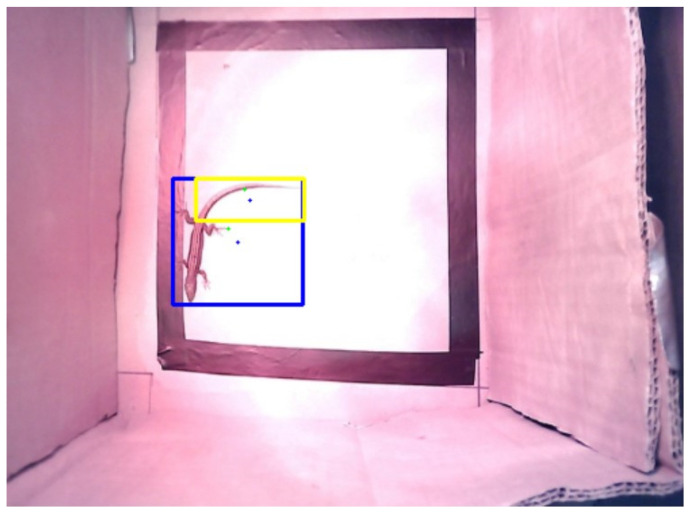
The initial pixel (centre) represents the “Lizard” (blue rectangle) and “Tail” (yellow rectangle) bounding boxes, marked with a blue dot. The final pixel representative of each bounding box is marked with a green dot.

In Figure 12, the green circle represents the closest white pixel found in the bounding box, starting from the central black pixel. These are the new pixels considered for the thermal analysis.

#### 2.2.4. Perspective Transformation and Temperature Detection

Perspective transformation is used to establish a relationship between pixels in the RGB image and corresponding pixels in the thermal image. In perspective transformation, a 3x3 transformation matrix is determined by four points in the RGB image and the corresponding four points in the thermal image. 

Using Python OpenCV’s library, Equation (7) calculates the transformation matrix.

matrix = cv2.getPerspectiveTransform(src, dst)(7)

where:

- The “src” parameter represents the coordinates of the quadrilateral vertices in the source image (RGB image).
- The “dst” parameter represents the coordinates of the corresponding quadrilateral vertices in the destination image (thermal image).

The parameters “src” and “dst” are defined by the function shown in Equation (8).

np.array([[x_min_, y_min_], [x_max_, y_min_], [x_max_, y_max_], [x_min_, y_max_]], dtype = np.float32) (8)

**Figure 13:**
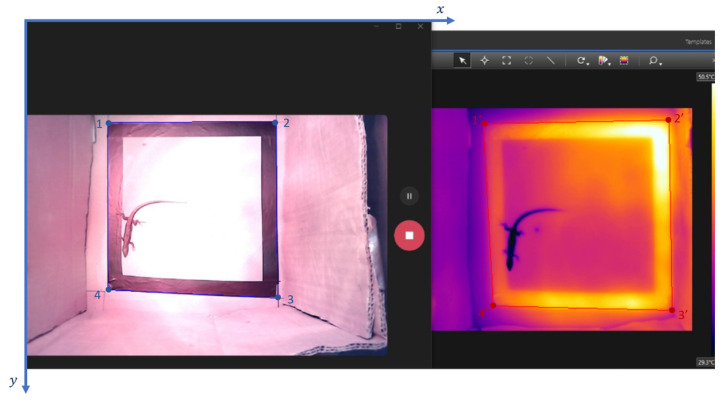
The “src” parameter is represented by the coordinates of points 1, 2, 3, and 4, and the “dst” parameter is represented by the coordinates of points 1′, 2′, 3′, and 4′.

According to Equation (8) and Figure 13, for the “src” parameter, [x_min_, y_min_] corresponds to the coordinates of point 1, [x_max_, y_min_] corresponds to the coordinates of point 2, [x_max_, y_max_] corresponds to the coordinates of point 3, and [x_min_, y_max_] corresponds to the coordinates of point 4. For the “dst” parameter, [x_min_, y_min_] corresponds to the coordinates of point 1′, [x_max_, y_min_] corresponds to the coordinates of point 2′, [x_max_, y_max_] corresponds to the coordinates of point 3′, and [x_min_, y_max_] corresponds to the coordinates of point 4′.

This way, it is possible to transform any set of coordinates using the transformation matrix. In Equation (9), the transformation matrix described in Equation (7) is applied to the point represented as [centre_x, centre_y, 1].

transf_coord = np.dot(matrix, np.array([centre_x, centre_y, 1], dtype = np.float32))(9)

Subsequently, the transformed coordinates obtained (“[x, y, w]”) in Equation (9) are normalised using Equation (10). In this equation, “x” and “y” are divided by “w”.

transf_coord = transf_coord[:2]/transf_coord [2](10)

In Equation (11), the normalised transformed coordinates are assigned to “xn” and “yn”.

xn,yn = tuple(transf_coord)(11)

In the Equations (12) and (13), “xn” and “yn” coordinates are rounded. “xn” and “yn” represent the final transformed coordinates in the thermal image corresponding to the original point in the RGB image (center_x, center_y).

xn = int(xn + 0.5)(12)

yn = int(yn + 0.5)(13)

**Figure 14:**
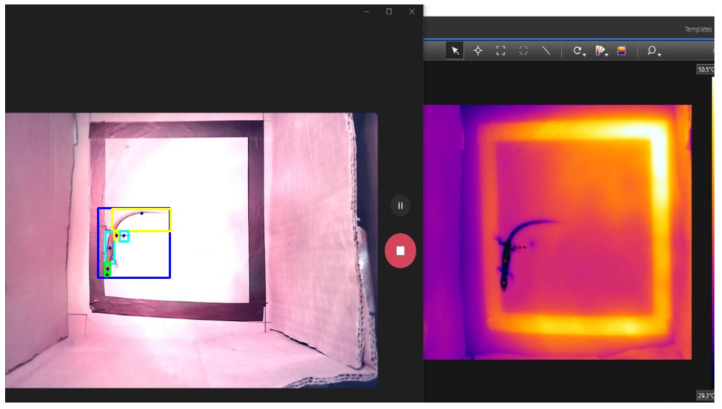
Bounding boxes and their representative pixels marked with blue dots (RGB image) and corresponding pixels marked with red dots in the thermal image.

After applying the equations mentioned above to each pixel marked in the RGB image (left side of Figure 14), it was possible to obtain the corresponding pixels in the thermal image (right side of Figure 14).

For each pixel marked in the thermal image, the respective temperature value was obtained through its colouring. It is important to highlight that the colour temperature scale can vary between images.

To make this possible, a function was created that allows obtaining the temperature based on a given pixel colour (input) and a set of parameters (T_max_, T_min_, Y_max_, Y_min_, X_med_). The temperature value is calculated using a linear interpolation, as shown in Equation (14), where “final” represents the row index.

(14) $$T=Tmin+\frac{\left(Y_{max}-final\right)}{\left(Y_{max}-Y_{min}\right)}\cdot (T_{max}-T_{min})$$

**Figure 15:**
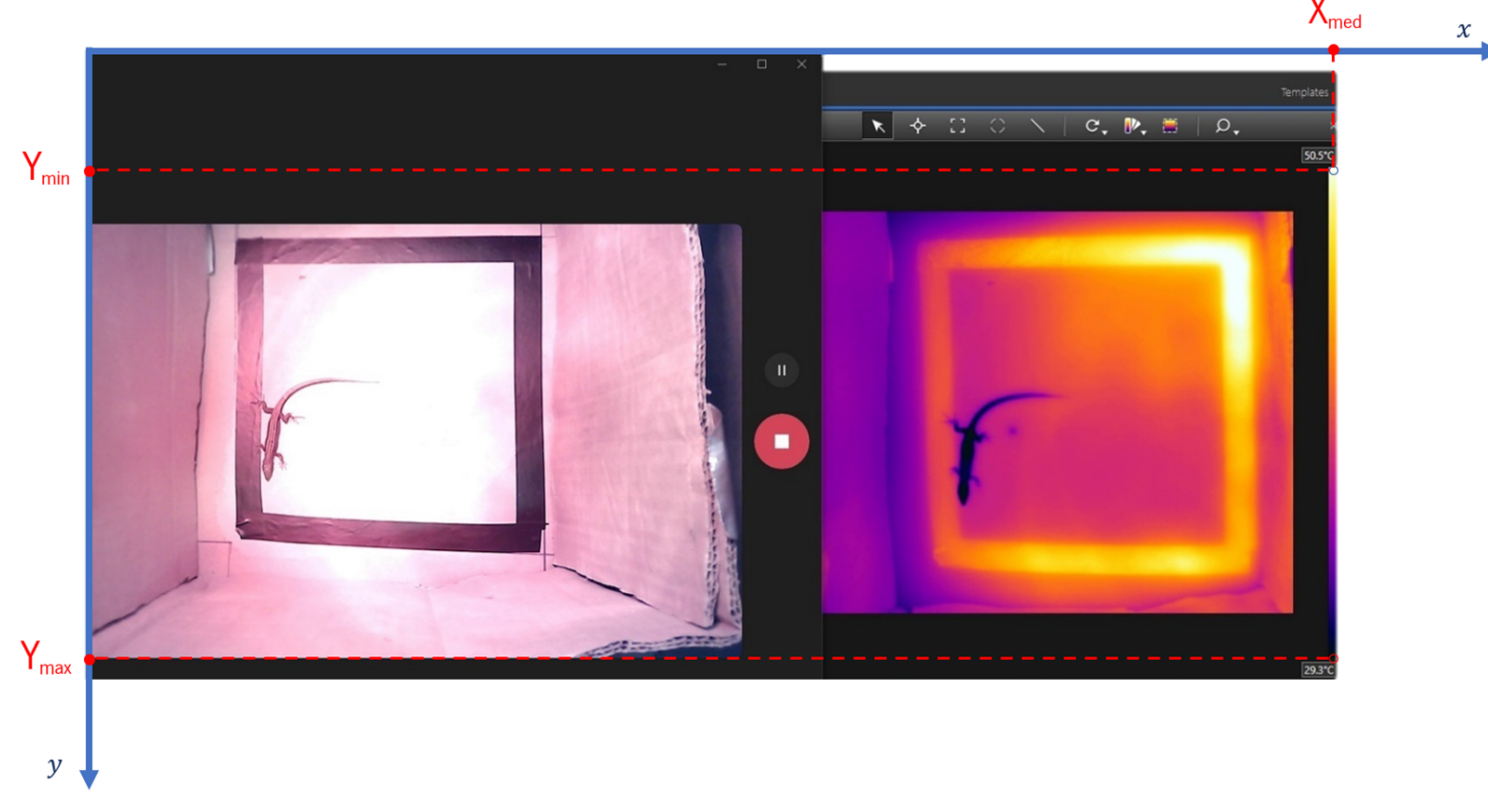
Annotation of the maximum Y (Y_max_), minimum Y (Y_min_), and median X (X_med_) relative to the coordinate axis of the input image.

The maximum temperature (T_max_), minimum temperature (T_min_), maximum Y (Y_max_), minimum Y (Y_min_), and median X (X_med_) values were defined based on the input image. Looking at the colour temperature scale present on the right side of Figure 15 (column of 10 pixels width represents the colour scale), it can be stated that the minimum temperature (T_min_) is 29.3 °C, and the maximum temperature (T_max_) is 50.5 °C. Also, the maximum Y (Y_max_) value corresponds to the y-coordinate of the bottom corner of the bar (for T_min_), the minimum Y (Y_min_) value corresponds to the y-coordinate of the top corner of the bar (for T_max_), and the median X (X_med_) value corresponds to the position of the bar on the x-axis.

The temperature values obtained for each class were automatically stored in a text file together with the day, time of measurements, and the class name.

## 3. Results and Discussion

This section contains the results of the training and inferences carried out to obtain the best model for detecting the lizard and its body parts. It also demonstrates the system’s potential in acquiring the temperature values of the parts detected by the model.

### 3.1. Detection of Lizard Body Parts: Training and Inference

The best training results were obtained using a batch size of 32 and 500 epochs for the neural network. This training took 15 h, 14 min, and 21 s.

Table 4 presents the values obtained for precision, recall, and mAP metrics for each of the seven classes. At the end, the average values of these metrics are shown.

By analysing Table 4, it is observed that the “Snout” class was the one that presented the lowest value in all the metrics. The reason for this may be due to the small size of this body part of the lizard in relation to the other parts, making its correct identification more complex.

Comparing the average values of mAP_0.5 and mAP_0.5:0.95 metrics, it is possible to perceive that the value of mAP_0.5:0.95 is significantly lower than mAP_0.5. This is common since, unlike mAP_0.5, mAP_0.5:0.95 evaluates the model over a wider range of IoU thresholds. The increasing of the IoU threshold results in stricter requirements, causing the mAP value to decrease; therefore, obtaining a high mAP_0.5:0.95 value can be challenging.

The average value obtained for mAP_0.5:0.95 (75.40%) can be considered good. Also, the average precision (99.00%), recall (98.80%), and mAP_0.5 (98.60%) values obtained are very good.

**Figure 16:**
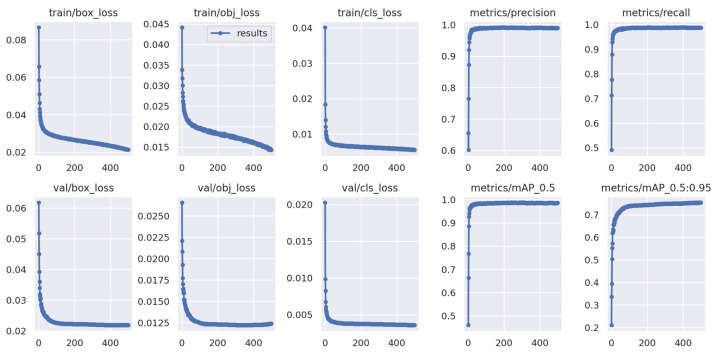
Resulting graphs after training using a batch size of 32 and 500 epochs.

Figure 16 displays all the graphs of the average values obtained after training. Each graph represents the change in a certain value (y-axis) as the number of epochs increases during training (x-axis). As mentioned previously, the number of epochs used in training was 500, so the x-axis will go up to the value of 500. The four graphs on the right side of Figure 16 correspond to the previously mentioned metrics: precision (“metrics/precision”), recall (“metrics/recall”), mAP_0.5 (“metrics/mAP_0.5”), and mAP_0.5:0.95 (“metrics/mAP_0.5:0.95”).

Observing the behaviour of the “metrics/precision”, “metrics/recall”, and “metrics/mAP_0.5” graphs, it is possible to understand that they begin to stabilise after about 80 epochs. The “metrics/mAP_0.5:0.95” graph started to stabilise later, after about 400 epochs.

The stabilisation of a graph indicates that there will no longer be significant improvements in the measured value. Therefore, to avoid the occurrence of overfitting and the decrease in metric values, the training was considered completed for the number of epochs of 500.

The remaining six graphs on the left side of Figure 16 represent the training losses (“train/box_loss”, “train/obj_loss”, and train/cls_loss”) and the validation losses (“val/box_loss”, “val/obj_loss”, and val/cls_loss”). Where “box_loss” is the box regression loss, “obj_loss” is the object loss, and “cls_loss” is the class loss. In these six graphs, it is possible to observe that, as desired, the loss values decreased as the number of epochs increased. Furthermore, a rapid decline was observed until around epoch 10.

By analysing the loss graphs in Figure 16, it can be concluded that overfitting did not occur.

**Figure 17:**
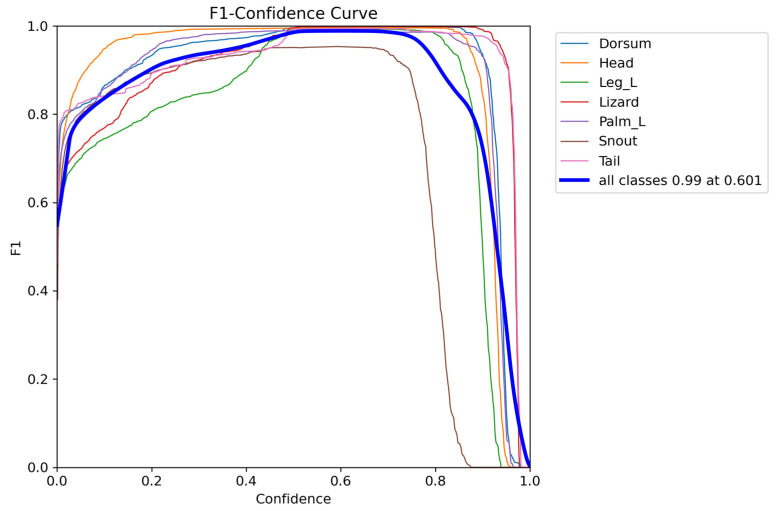
F1–confidence curve.

The F1 score curve illustrates the F1 score across different thresholds, offering insights into the model’s balance between false positives and false negatives. Figure 17 shows that the maximum F1 value is 0.99 when the confidence score is 0.601.

**Figure 18:**
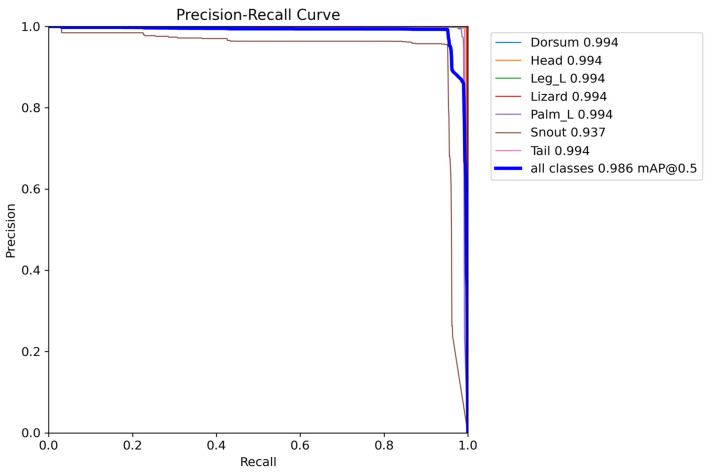
Precision–recall curve.

Figure 18 presents the precision–recall curve, where a larger area under the curve indicates better overall performance. The “Snout” class has a smaller area under the PR curve, indicating that the model has more difficulty in correctly detecting this class compared to the others. (mAP_0.5 of 0.937).

**Figure 19:**
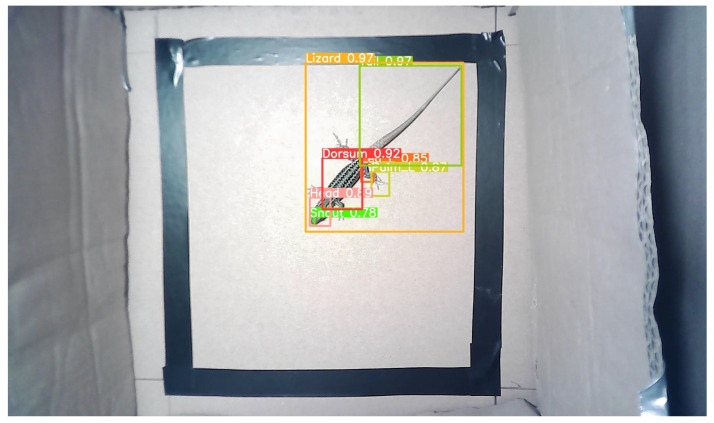
Example of an image from the “test set” with predictions.

To evaluate how well the trained model generalises to unseen images, the inference was run on the images from the “test set”. Figure 19 shows a sample image used in the inference. As expected, the predictions were acceptable. All classes were correctly indicated with confidence scores ranging from 78% to 97%.

**Figure 20:**
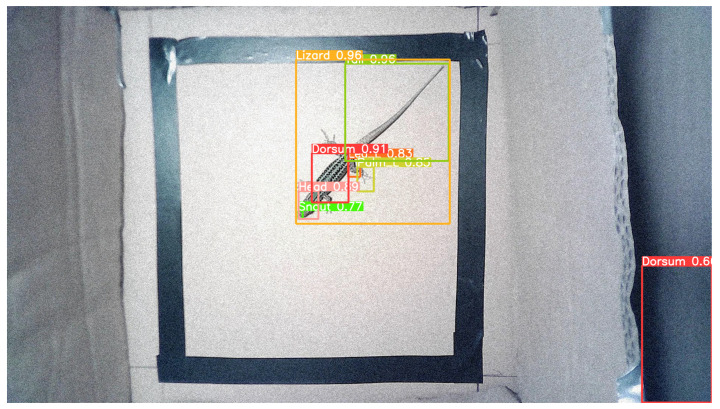
Example of an image from the “test set” with noise and predictions.

Noise was added to Figure 19, as demonstrated in Figure 20, to analyse the model’s performance on noisy images and variations in image quality. As shown in Figure 20, the model was able to correctly detect the lizard and its six body parts with confidence scores ranging from 77% to 96%. However, one more bounding box corresponding to the “Dorsum” class was incorrectly detected, with a confidence score of 60%.

When the model is faced with cases for which it was not trained, it tends to show a decrease in the confidence score of the detected classes and may even generate false positives.

### 3.2. Temperature Acquisition

**Figure 21:**
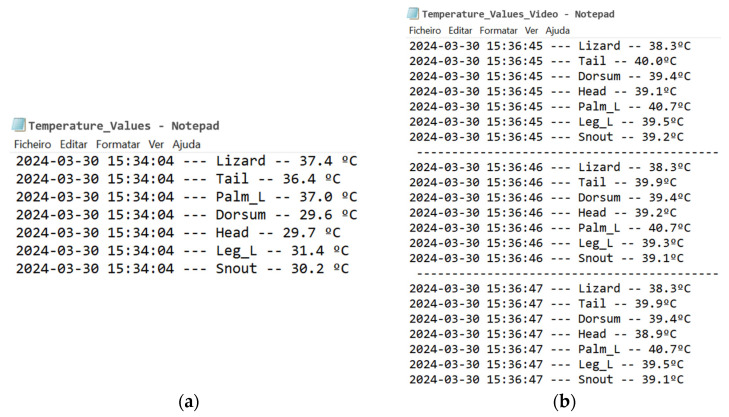
Notepad with date, hour, class, and temperature values obtained: (**a**) from an image and (**b**) from a video.

Applying the methodologies presented in Section 2.2, it was possible to successfully obtain the final temperature values in different images and videos. Figure 21 shows an example of the temperature values obtained for the lizard and its body parts in an image and a video.

The accuracy of temperature measurements is given by the thermal camera used; in this case, the FLIR T335 thermal camera, which has an accuracy of ±2 °C of the reading.

### 3.3. Comparison with Other Studies for Automatic Detection and Temperature Extraction

In recent years, several studies have been carried out to develop methods for automatically detecting specific body parts of animals and extracting their body temperature values. These efforts were driven by the need to address the limitations and challenges associated with traditional manual temperature measurement techniques.

Xie et al. [37] developed an automatic temperature detection method based on Infrared Thermography (ITG) to overcome the challenges associated with traditional pig rectal temperature measurement. Automatic detection of six regions on the pig body surface (forehead, eyes, nose, ear root, back, and anus) was performed using an improved YOLOv5s model with BiFPN. After detection, the temperature values were automatically extracted. The proposed YOLOv5s-BiFPN model achieved optimal performance, with a mAP of 96.36%, a target detection speed of up to 100 frames per second, and a model size of 20 MB. Additionally, the variations in maximum temperature automatically extracted from the ear root and the forehead coincided with those obtained manually, and the temperature accuracy was ±2 °C.

Wang et al. [38] proposed a method based on the detection model GG-YOLOv4 for the automatic detection of the ocular surface temperature of dairy cows from thermal images, with the aim of identifying health disorders. The model achieved a mAP of 96.88%, a detection speed of 40.33 frames per second, and a model size of 44.7 M. The comparison between the temperature values obtained with the model and the manually extracted values showed that the average absolute temperature extraction errors in the left and right eyes were 0.051 °C and 0.042 °C, respectively, and the average relative temperature extraction errors in the left and right eyes were 0.14% and 0.11%, respectively. The temperature accuracy was ±2 °C.

The proposed model in this paper achieved a mean average precision (mAP) of 98.60%, outperforming the models developed by Xie et al. [37] and Wang et al. [38]. All methods mentioned above have the same temperature accuracy value (±2 °C).

The algorithm proposed in this paper introduces innovative features to improve the detection of lizards and the extraction of their body temperature in a controlled laboratory environment. Firstly, this study significantly contributes to filling the notable gap in algorithm development and research regarding automatic and non-invasive methods for lizard detection and body temperature extraction in controlled laboratory environments. Secondly, employing a non-invasive and automatic method for extracting the body temperature of lizards in a controlled laboratory environment minimises potential harm and stress to the animals, thereby promoting a more efficient and humane way of monitoring lizard body temperature. Thirdly, due to the scarcity of publicly available lizard datasets, a dataset was created from scratch, providing a valuable resource for training and potentially benefiting future lizard-related research. Lastly, the simultaneous use of two cameras (RGB and thermal camera) significantly enhances the accuracy of lizard detection and enables precise temperature extraction.

In the dual-camera system, the RGB camera allows the detection of the lizard and its body parts using YOLOv5, and the thermal camera allows reading of the respective temperature of those parts. After calibration, the images from both cameras are properly localised, making it possible to determine accurately the lizard’s position in the thermal image through a coordinate conversion from the RGB image to the thermal image. Based on the colour temperature scale present in the thermal image, the temperature values are then extracted. Therefore, this approach enables the automated and non-invasive extraction of the lizard’s body temperature.

## 4. Conclusions

The work presented in this paper concerns the development of a system capable of detecting the lizard and its body parts, subsequently acquiring their respective temperature values. This method provides biologists with a faster and non-intrusive way to measure lizard body temperature in a controlled laboratory setting, allowing researchers to monitor the temperature preferences of lizards and enabling detailed observations of their thermoregulation strategies. By automating the temperature acquisition process, this method reduces stress and potential harm to the animals, offering a more ethical approach to studying lizards’ behaviour.

This work can be divided into two main parts: the dataset creation and the detection of the lizard and its body parts; and the acquisition of the respective temperature values.

Since there were no datasets available online or in the Biology Laboratory, it was necessary and challenging to create a dataset from scratch, including creating a scenario, filming videos, obtaining frames from these videos, and labelling the images with each class.

The YOLOv5s (small) model was chosen because it is lightweight, has a fast inference time, and offers the best balance between training duration and the quality of the results obtained. When using the model to detect the lizard and its body parts, challenges were encountered in more complex images (images with noise), leading to some classes being incorrectly detected. However, the model correctly identified the lizard and its body parts in all images from the “test set”, with confidence scores above 78% in which, in general, the “Lizard” (average of 96%) and “Tail” (average of 94%) classes presented the highest confidence scores, and the “Snout” class was the one with the lowest confidence score (average of 78%). The model achieved a precision of 90.00% and a recall of 98.80%. It can be concluded that the application of YOLOv5s for the detection of lizards and their body parts has demonstrated overall success.

The model was used to make detections only in RGB images and not in thermal images since it was trained only with RGB images. If the model was used in thermal images, it would generate erroneous detections because sometimes the lizard is not visible in the thermal image due to its temperature being equal to its background floor. The coordinate transformation from the RGB image to the thermal image proved to be effective, allowing the acquisition of the final temperature values of the lizard’s body parts based on the colour temperature scale and the colour of the pixels present in the thermal image. The accuracy in acquiring the temperature values directly relied on the precise mapping of coordinates between the RGB and thermal images.

Overall, the system successfully achieves the intended end goal. However, it is important to highlight that there is still room for improvement.

Given the challenges encountered during the development of this work and the respective results obtained, a few proposals are presented to be implemented in future updates.

- Adaptation of the developed system to detect the body temperature of another species of animal kept in captivity.
- RGB and thermal cameras with better resolution.
- Obtain the values of additional parameters, such as emissivity and reflective temperature, that allow acquiring new information regarding the temperature measurement process, enabling a deeper analysis.

## Figures and Tables

**Table 1:** Values obtained for precision, recall, mAP, training duration, number of parameters, GFLOPs, and inference time using YOLOv5n and YOLOv5s.

Metrics	YOLOv5n	YOLOv5s
Precision (%)	98.60	99.00
Recall (%)	97.60	98.40
mAP_0.5 (%)	97.90	98.40
mAP_0.5:0.95 (%)	71.50	74.30
Training Duration	2 h 18 min 22 s	3 h 22 min 34 s
Parameters (M)	1.8	7.0
GFLOPs	4.2	15.8
Inference Time (ms)	5.5	9.9

**Table 2:** Values obtained for precision, recall, mAP, training duration, number of parameters, GFLOPs, and inference time using YOLOv5m, YOLOv5l, and YOLOv5x.

Metrics	YOLOv5m	YOLOv5l	YOLOv5x
Precision (%)	99.10	99.10	99.10
Recall (%)	98.70	98.90	99.00
mAP_0.5 (%)	98.70	98.80	98.90
mAP_0.5:0.95 (%)	76.10	76.20	76.30
Training Duration	6 h 36 min 8 s	10 h 39 min 19 s	17 h 32 min 47 s
Parameters (M)	2.1	4.6	86.2
GFLOPs	47.9	107.7	203.9
Inference Time (ms)	13.1	25.0	47.8

**Table 3:** Number of epochs and batch sizes used for training.

Batch	Epoch
16	100
	200
20	100
	200
32	100
	200
	300
	400
	500
64	100
	200
	300
	400

**Table 4:** Values obtained for precision, recall, and mAP after training using a batch size of 32 and a number of epochs of 500.

Class	Precision (%)	Recall (%)	mAP_0.5 (%)	mAP_0.5:0.95 (%)
Lizard	99.80	99.90	99.40	92.20
Snout	95.60	95.00	93.70	42.70
Head	99.50	99.60	99.40	74.50
Dorsum	99.70	99.90	99.40	78.70
Tail	99.60	99.20	99.40	94.50
Leg_L	99.50	99.20	99.40	66.80
Palm_L	99.40	98.40	99.40	78.60
AVERAGE	99.00	98.80	98.60	75.40

## Data Availability

The original data presented in the study are openly available in OSF at DOI 10.17605/OSF.IO/UQYEG.

## References

[1] Jumper J., Evans R., Pritzel A., Green T., Figurnov M., Ronneberger O., Tunyasuvunakool K., Bates R., Žídek A., Potapenko A. (2021). Highly Accurate Protein Structure Prediction with AlphaFold. Nature.

[2] Jiménez-Luna J., Grisoni F., Schneider G. (2020). Drug Discovery with Explainable Artificial Intelligence. Nat. Mach. Intell..

[3] Buchelt A., Adrowitzer A., Kieseberg P., Gollob C., Nothdurft A., Eresheim S., Tschiatschek S., Stampfer K., Holzinger A. (2024). Exploring Artificial Intelligence for Applications of Drones in Forest Ecology and Management. For. Ecol. Manag..

[4] Hurwitz J., Kirsch D. (2018). Understanding Machine Learning. Machine Learning for Dummies.

[5] Mueller J.P., Massaron L. (2021). Descending the Gradient. Machine Learning for Dummies.

[6] Burkov A. (2019). Basic Practice. The Hundred-Page Machine Learning Book.

[7] Cunningham P., Cord M., Delany S.J., Cord M., Cunningham P. (2008). Supervised Learning. Machine Learning Techniques for Multimedia.

[8] Lecun Y., Bottou L., Bengio Y., Haffner P. (1998). Gradient-Based Learning Applied to Document Recognition. Proc. IEEE.

[9] Krizhevsky A., Sutskever I., Hinton G.E. (2017). ImageNet Classification with Deep Convolutional Neural Networks. Commun. ACM.

[10] Zeiler M.D., Fergus R., Fleet D., Pajdla T., Schiele B., Tuytelaars T. (2014). Visualizing and Understanding Convolutional Networks. Computer Vision–ECCV 2014.

[11] Szegedy C., Liu W., Jia Y., Sermanet P., Reed S., Anguelov D., Erhan D., Vanhoucke V., Rabinovich A. Going Deeper with Convolutions. Proceedings of the 2015 IEEE Conference on Computer Vision and Pattern Recognition (CVPR).

[12] He K., Zhang X., Ren S., Sun J. Deep Residual Learning for Image Recognition. Proceedings of the 2016 IEEE Conference on Computer Vision and Pattern Recognition (CVPR).

[13] Simonyan K., Zisserman A. (2015). Very Deep Convolutional Networks for Large-Scale Image Recognition. 3rd International Conference on Learning Representations (ICLR 2015).

[14] Tan M., Le Q. (2019). EfficientNet: Rethinking Model Scaling for Convolutional Neural Networks. Proceedings of the 36th International Conference on Machine Learning.

[15] Liu W., Anguelov D., Erhan D., Szegedy C., Reed S., Fu C.-Y., Berg A.C., Leibe B., Matas J., Sebe N., Welling M. (2016). SSD: Single Shot MultiBox Detector. Computer Vision—ECCV 2016.

[16] Lin T.-Y., Goyal P., Girshick R., He K., Dollar P. Focal Loss for Dense Object Detection. Proceedings of the 2017 IEEE International Conference on Computer Vision (ICCV).

[17] Tan M., Pang R., Le Q.V. EfficientDet: Scalable and Efficient Object Detection. Proceedings of the 2020 IEEE/CVF Conference on Computer Vision and Pattern Recognition (CVPR).

[18] Hao M., Sun Q., Xuan C., Zhang X., Zhao M., Song S. (2024). Lightweight Small-Tailed Han Sheep Facial Recognition Based on Improved SSD Algorithm. Agriculture.

[19] Jia J., Fu M., Liu X., Zheng B. (2022). Underwater Object Detection Based on Improved EfficientDet. Remote Sens..

[20] Roy A.M., Bhaduri J., Kumar T., Raj K. (2023). WilDect-YOLO: An Efficient and Robust Computer Vision-Based Accurate Object Localization Model for Automated Endangered Wildlife Detection. Ecol. Inform..

[21] Hu J., Jagtap R., Ravichandran R., Sathya Moorthy C.P., Sobol N., Wu J., Gao J. (2023). Data-Driven Air Quality and Environmental Evaluation for Cattle Farms. Atmosphere.

[22] Jubayer F., Soeb J.A., Mojumder A.N., Paul M.K., Barua P., Kayshar S., Akter S.S., Rahman M., Islam A. (2021). Detection of Mold on the Food Surface Using YOLOv5. Curr. Res. Food Sci..

[23] Long W., Wang Y., Hu L., Zhang J., Zhang C., Jiang L., Xu L. (2024). Triple Attention Mechanism with YOLOv5s for Fish Detection. Fishes.

[24] Ahmad I., Yang Y., Yue Y., Ye C., Hassan M., Cheng X., Wu Y., Zhang Y. (2022). Deep Learning Based Detector YOLOv5 for Identifying Insect Pests. Appl. Sci..

[25] Su X., Zhang J., Ma Z., Dong Y., Zi J., Xu N., Zhang H., Xu F., Chen F. (2024). Identification of Rare Wildlife in the Field Environment Based on the Improved YOLOv5 Model. Remote Sens..

[26] Qiao Y., Guo Y., He D. (2023). Cattle Body Detection Based on YOLOv5-ASFF for Precision Livestock Farming. Comput. Electron. Agric..

[27] Jiang B., Wu Q., Yin X., Wu D., Song H., He D. (2019). FLYOLOv3 Deep Learning for Key Parts of Dairy Cow Body Detection. Comput. Electron. Agric..

[28] Tannous M., Stefanini C., Romano D. (2023). A Deep-Learning-Based Detection Approach for the Identification of Insect Species of Economic Importance. Insects.

[29] Hamzaoui M., Ould-Elhassen Aoueileyine M., Romdhani L., Bouallegue R. (2023). An Improved Deep Learning Model for Underwater Species Recognition in Aquaculture. Fishes.

[30] Aota T., Ashizawa K., Mori H., Toda M., Chiba S. (2021). Detection of Anolis Carolinensis Using Drone Images and a Deep Neural Network: An Effective Tool for Controlling Invasive Species. Biol. Invasions.

[31] Guo S.-S., Lee K.-H., Chang L., Tseng C.-D., Sie S.-J., Lin G.-Z., Chen J.-Y., Yeh Y.-H., Huang Y.-J., Lee T.-F. (2022). Development of an Automated Body Temperature Detection Platform for Face Recognition in Cattle with YOLO V3-Tiny Deep Learning and Infrared Thermal Imaging. Appl. Sci..

[32] Zhang B., Xiao D., Liu J., Huang S., Huang Y., Lin T. (2024). Pig Eye Area Temperature Extraction Algorithm Based on Registered Images. Comput. Electron. Agric..

[33] Karameta E., Gavriilidi I., Sfenthourakis S., Pafilis P. (2023). Seasonal Variation in the Thermoregulation Pattern of an Insular Agamid Lizard. Animals.

[34] Liu S., Qi L., Qin H., Shi J., Jia J. Path Aggregation Network for Instance Segmentation. Proceedings of the 2018 IEEE/CVF Conference on Computer Vision and Pattern Recognition.

[35] Wang C.-Y., Mark Liao H.-Y., Wu Y.-H., Chen P.-Y., Hsieh J.-W., Yeh I.-H. CSPNet: A New Backbone That Can Enhance Learning Capability of CNN. Proceedings of the 2020 IEEE/CVF Conference on Computer Vision and Pattern Recognition Workshops (CVPRW).

[36] Terven J., Córdova-Esparza D.-M., Romero-González J.-A. (2023). A Comprehensive Review of YOLO Architectures in Computer Vision: From YOLOv1 to YOLOv8 and YOLO-NAS. Mach. Learn. Knowl. Extr..

[37] Xie Q., Wu M., Bao J., Zheng P., Liu W., Liu X., Yu H. (2023). A Deep Learning-Based Detection Method for Pig Body Temperature Using Infrared Thermography. Comput. Electron. Agric..

[38] Wang Y., Kang X., Chu M., Liu G. (2022). Deep Learning-Based Automatic Dairy Cow Ocular Surface Temperature Detection from Thermal Images. Comput. Electron. Agric..

